# Human β-defensin 3 affects the activity of pro-inflammatory pathways associated with MyD88 and TRIF

**DOI:** 10.1002/eji.201141648

**Published:** 2011-08-02

**Authors:** Fiona Semple, Heather MacPherson, Sheila Webb, Sarah L Cox, Lucy J Mallin, Christine Tyrrell, Graeme R Grimes, Colin A Semple, Matthew A Nix, Glenn L Millhauser, Julia R Dorin

**Affiliations:** 1 MRC Human Genetics UnitIGMM, Edinburgh, Scotland, UK; 2 Department of Chemistry, University of CaliforniaSanta Cruz, CA, USA

**Keywords:** Anti-inflammatory, Defensin, Innate immunity, Microarray, Toll-like receptor

## Abstract

β-Defensins are cationic host defense peptides that form an amphipathic structure stabilized by three intramolecular disulfide bonds. They are key players in innate and adaptive immunity and have recently been shown to limit the production of pro-inflammatory cytokines in TLR4-stimulated macrophages. In the present study, we investigate the mechanism underlying the anti-inflammatory effect of human β-defensin 3 (hBD3). We show that the canonical structure of hBD3 is required for this immunosuppressive effect and that hBD3 rapidly associates with and enters macrophages. Examination of the global effect of hBD3 on transcription in TLR4-stimulated macrophages shows that hBD3 inhibits the transcription of pro-inflammatory genes. Among the altered genes there is significant enrichment of groups involved in the positive regulation of NF-κB including components of Toll-like receptor signaling pathways. We confirm these observations by showing corresponding decreases in protein levels of pro-inflammatory cytokines and cell surface molecules. In addition, we show that hBD3 reduces NF-κB signaling in cells transfected with MyD88 or TRIF and that hBD3 inhibits the TLR4 response in both MyD88- and TRIF-deficient macrophages. Taken together these findings suggest that the mechanism of hBD3 anti-inflammatory activity involves specific targeting of TLR signaling pathways resulting in transcriptional repression of pro-inflammatory genes.

## Introduction

The mammalian immune system has developed a multitude of mechanisms to deal with invading micro-organisms. β-Defensins are cationic, antimicrobial peptides believed to defend the host against invading microbes by inhibiting bacterial cell wall biosynthesis 1. In addition, β-defensins have immunomodulatory properties and chemoattract monocytes, lymphocytes and DCs 2, suggesting that these antimicrobial peptides play a role in the link between innate and adaptive immunity. Human β-defensins chemoattract immune cells and hBD3 in particular has been shown to bind a variety of receptors including CCR6, CCR2 3, 4 melanocortin receptors 5 and the chemokine receptor CXCR4 6.

β-Defensins have been shown to increase expression of pro-inflammatory mediators in keratinocytes 7 and immune cells. In immature DCs, murine DEFB2 has been described as a ligand for Toll-like receptor 4 (TLR4) and induces maturation and co-stimulatory molecules 8. In addition, hBD3 has been shown to induce expression of co-stimulatory molecules CD80, CD86 and CD40, on human monocytes and myeloid DCs in a TLR1/2-dependent manner 9. Conversely, some studies have described an inhibitory effect of defensins. DEFB123 prevents LPS-mediated effects in vitro and in vivo 10, and a range of α- and β-defensins have significantly inhibited the expression of LPS-induced pro-inflammatory cytokines 11, 12. In some cases the researchers attribute the suppressive effects of defensins on the immune response to defensin-LPS binding 10, 12 as has previously been described for the bactericidal/permeability increasing protein from granulocytes 13 and the cathelicidin LL-37 14.

We previously described an anti-inflammatory role of hBD3 by demonstrating that hBD3 inhibits the accumulation of TNF-α and IL-6 proteins in LPS-stimulated BM-derived macrophages both in vitro and in vivo 15. The mechanism of this activity is unknown and it may be that, like other defensins 8, 10, hBD3 is binding LPS in the extracellular space. hBD3 may also bind to TLR4 thereby preventing the TLR4-LPS interaction. However, we have previously shown that hBD3 does not bind LPS in the limulus amebocyte assay and that hBD3 also inhibits a non-TLR ligand, CD40L 15. In the present study, we further clarify the mechanism of hBD3 anti-inflammatory action by examining the requirement for structural integrity; the uptake of hBD3 by macrophages; the transcriptional response of macrophages and the intracellular signaling pathways suppressed by hBD3.

## Results

### The importance of the structural integrity of hBD3

**Figure 1:**
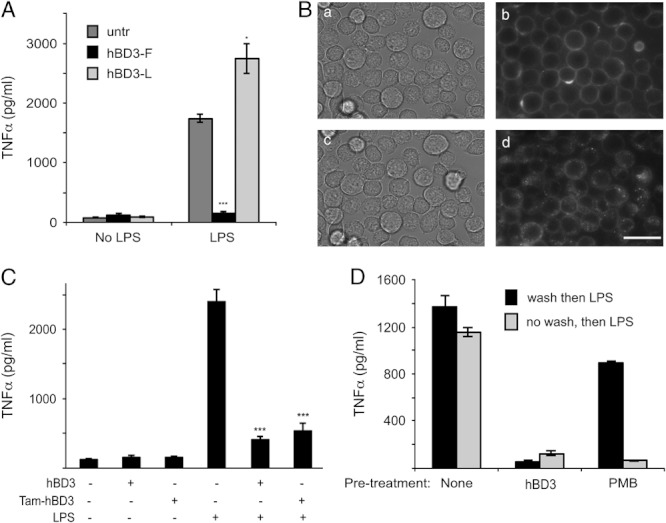
hBD3 anti-inflammatory effect is dependent on structure and cellular uptake. (A) RAW264.7 cells were treated with 50 ng/mL LPS with and without either canonically folded hBD3 (hBD3-F) or linearised hBD3 (hBD3-L) each at 5 μg/mL for 4 h. TNF-α in tissue culture supernatant was measured by ELISA, *n*=3. Data are presented as means±SEM, significance assessed by unpaired *t*-test, ^***^p<0.001, ^*^p<0.05 was calculated by comparing LPS plus hBD3 with LPS alone. (B) RAW 264.7 cells cultured on glass overnight were exposed to 5 μg/mL hBD3^TAMRA^. A z-series was collected for each treatment with an interplane distance of 0.2 μM. The optimal *z*-plane from each stack is shown. The fluorescent images show the cells immediately (b) and 10 mins (d) after exposure to the peptide, with the corresponding brightfield images shown in (a) and (c). Scale bar 10 μM. (C) TNF-α in tissue culture supernatant from RAW264.7 treated as above, was measured by ELISA, *n*=3. Data are presented as means±SEM, significance assessed by unpaired *t*-test, ^***^p<0.001, was calculated by comparing LPS plus hBD3 or hBD3^TAMRA^, with LPS alone. (D) RAW264.7 cells were pre-treated in serum-free media with 5 μg/mL hBD3 or 10 μg/mL polymyxin B for the indicated times, washed twice with PBS, then returned to conditioned serum-free media before incubation with 50 ng/mL LPS for 4 h. TNF-α in tissue culture supernatant from was measured by ELISA, *n*=3. Data are presented as means±SEM, significance assessed by unpaired *t*-test, ^***^p<0.001, was calculated by comparing LPS plus hBD3 with LPS alone.

We have previously shown that hBD3 does not induce a pro-inflammatory response, but suppresses the cytokine response of macrophages to LPS. Other researchers however, have found hBD3 to have pro-inflammatory effects in immune cells 9. We investigated whether the structural integrity of hBD3 plays an important part in the immune-modulating effect of this peptide. Structure has previously been shown to affect hBD3–receptor interactions and chemotactic activity 16. hBD3 has an N-terminal α-helix followed by three antiparallel β sheets, which is stabilized by six canonical cysteines and three disulfide bonds 17. To determine whether the disulfide bond stabilization of hBD3 is important for the anti-inflammatory effect, we validated the oxidation of our hBD3 preparation and also synthesized a form of hBD3 where the six cysteine residues were replaced by serines (hBD3cys-ser). This peptide has the same net charge and hydrophobicity as the parent peptide. hBD3cys-ser did not inhibit LPS-induced TNF-α and in fact significantly enhanced the pro-inflammatory effects of LPS, in contrast to the anti-inflammatory effects observed with folded hBD3 (Fig. 1A). In the absence of LPS, hBD3cys-ser had no effect on TNF-α levels. None of the above treatments affected cell viability as MTT assay measurements were comparable between treated cells and untreated controls. These data suggest that hBD3, in both structural forms, does not induce a pro-inflammatory response, but in the presence of LPS, hBD3 that is not canonically folded enhances the pro-inflammatory effects of LPS in macrophages.

### hBD3 rapidly enters macrophages

To investigate whether hBD3 remains at the cell surface or enters the cell, we treated RAW264.7 macrophage cells with TAMRA-labeled hBD3 (hBD3^TAMRA^) and used live cell imaging to track uptake of the labeled peptide. Immediately after addition, hBD3^TAMRA^ was evident on the surface of the cell membrane (Fig. 1B, panel b). After 10 mins labeled hBD3 was visualized in the cell cytoplasm, and did not appear to enter the nucleus (Fig. 1B, panel d). hBD3^TAMRA^ continued to accumulate both inside the cell and on the cell surface over 2 h (Supporting Information Movie) whereas TAMRA label alone did not enter cells over a period of 24 h (Supporting Information Fig. 1). The presence of the TAMRA label did not affect the anti-inflammatory properties of hBD3 (Fig. 1C).

To demonstrate that the anti-inflammatory effect of hBD3 was not simply due to hBD3-LPS binding, we treated RAW264.7 cells with hBD3 for 1 h and washed the cells thoroughly before stimulating with LPS. Removal of hBD3 from the extracellular environment did not alter the ability of hBD3 to inhibit the pro-inflammatory effects of LPS (Fig. 1D). In comparison, the anti-inflammatory effect of polymyxin B, a cyclic hydrophobic peptide known to bind LPS, was significantly affected by washing. These results in combination with our previous finding that hBD3 significantly inhibits the effects of LPS, even when cells are treated with LPS for 1 h before the addition of hBD3, strongly suggests that most of the hBD3 inhibitory effect is occurring downstream of TLR4 activation by LPS.

### hBD3 represses TLR4-mediated transcription

**Figure 2:**
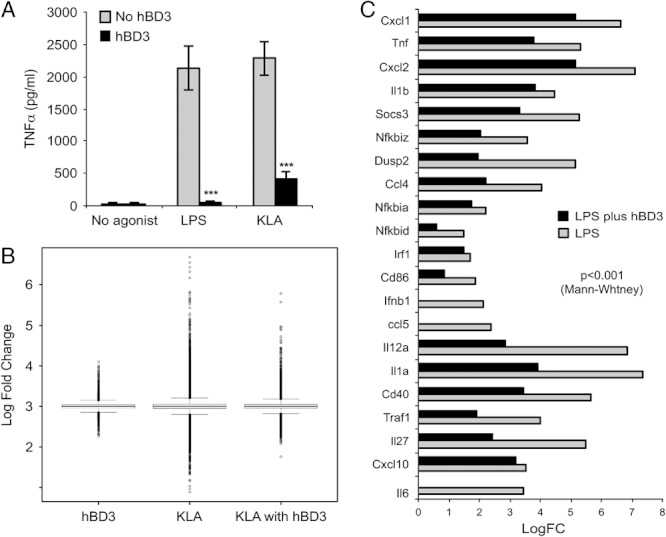
hBD3 inhibits LPS/KLA induced transcription. (A) BMDMs were treated with 50 ng/mL LPS or KLA in the presence and absence of 5 μg/mL hBD3 for 18 h. TNF-α in the supernatant was measured by ELISA, *n*=3 (BMDMs from three separate mice). Data are presented as means±SEM, significance assessed by unpaired *t*-test, ^***^p<0.001 was calculated by comparing LPS or KLA plus hBD3 with LPS or KLA alone. (B) Global gene expression changes in BMDMs treated for 6 h with 50 ng/mL KLA in the presence and absence of 5 μg/mL hBD3. Each box represents the interquartile range centered upon the median (and containing 50% of the values), the whiskers denote 1.5 times the interquartile range, and the circles represent outlier values beyond the whiskers. For each treatment (*x*-axis) the distribution of log FC values (*y*-axis) for all genes on the array are shown relative to untreated cells. Each circle on the graph represents a gene on the array. (C) Examples of genes suppressed by hBD3. The genes differentially expressed after BMDM stimulation with KLA (striped bars) were compared to the genes differentially expressed after BMDM stimulation in the presence of hBD3 (black bars). Each bar represents the average log fold change value compared to untreated BMDMs. Each treatment has been carried out in triplicate on three separate mice. *p*<0.001 Mann–Whitney test.

To further investigate the molecular mechanisms underlying the hBD3 effect, we profiled global gene expression changes in macrophages. A chemically defined LPS molecule, which consists of lipid A with an attached 3-deoxy-d-manno-octulosonic acid, termed KDO_2_-Lipid A (KLA) has been shown to induce comparable bioactivity and displays a similar gene expression profile to wildtype LPS from Gram-negative bacteria 18. We compared the effects of hBD3 on LPS- and KLA-stimulated BMDM and found that hBD3 significantly inhibited TNF-α induced by both LPS and KLA (Fig. 2A). To examine the effects of hBD3 on gene expression, BMDM from Balb/c mice were treated with KLA in the presence and absence of hBD3, hBD3 alone or left untreated. Previous studies have shown a marked increase in the transcription of pro-inflammatory cytokines at 6 h 19, we therefore examined patterns of expression associated with hBD3 and/or KLA treatment at 6 h.

As expected the analysis of the microarray data revealed evidence of significant expression changes in response to KLA. Compared with unstimulated macrophages, 6 h KLA treatment resulted in 5494 differentially expressed genes (multiple testing adjusted *p*-value of <0.05). This list of genes (Supporting Information Fig. 2, list 1) contained many genes reported to be altered in LPS-stimulated macrophages 20 and we observed a significant positive correlation (Pearson's *r*=0.585, *p*<0.01) with the LPS gene expression profile presented by Amit et al. 21.

Exposing macrophages to a combination of KLA and hBD3 dramatically reduced the number of genes significantly differentially expressed, from 5494 to 1779 (Supporting Information Fig. 2, list 2). We examined the distributions of expression changes, as log fold change (log FC) values, for all genes on the array between untreated cells and two treatments: KLA and KLA plus hBD3. The changes in distributions revealed a dramatic reduction in the spread of log FC values in the presence of hBD3, consistent with hBD3 globally attenuating the transcriptional response to KLA (Fig. 2B). We used the bioinformatics tool DAVID 22, 23 and identified significant KEGG (Kyoto Encyclopedia of Genes and Genomes) pathway enrichment among the genes differentially expressed between KLA treatment and KLA treatment in the presence of hBD3. Many of the genes suppressed by hBD3 are involved in both the TLR signaling and NOD-like receptor signaling pathways (Supporting Information Table 1). The genes demonstrating the highest log FC values after KLA treatment alongside the corresponding decreases in expression with treatment in the presence of hBD3 are shown in Fig. 2C. The expression values for KLA treatment are significantly different from log FC values for combined KLA and hBD3 treatment (*p*<0.001, Mann-Whitney test). These data suggest that hBD3 limits the effects of TLR4 stimulation by KLA by inhibiting gene transcription and/or events leading to gene transcription.

### hBD3 repression of transcription is reflected by a reduction in protein levels

**Figure 3:**
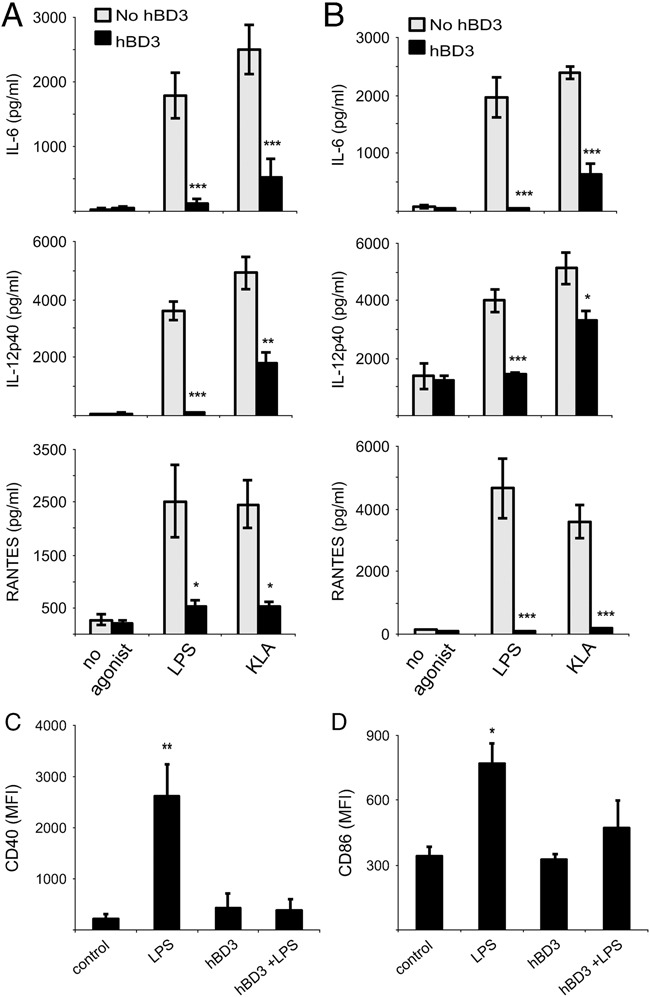
hBD3 inhibits production of TLR4-induced IL-6, IL-12p40, RANTES and co-stimulatory molecules. (A) BMDMs and (B) cDCs were treated with 50 ng/mL LPS or KLA in the presence or absence of 5 μg/mL hBD3 for 18 h. Cytokine levels in the cell culture supernatants were measured by ELISA, *n*=3 (BMDMs from three separate mice and cDCs from three further separate mice). Data are presented as means±SEM, significance assessed by unpaired *t*-test, ^***^p<0.001, ^**^p<0.01, ^*^p<0.05 was calculated by comparing LPS or KLA plus hBD3 with LPS or KLA alone. (C, D) BMDM were treated with 50 ng/mL LPS in the presence and absence of 5 μg/mL hBD3. Cell surface expression of (C) CD40 and (D) CD86 was assessed by flow cytometry. Data are presented as mean fluorescence intensity (MFI) demonstrating a significant increase in CD40 (^**^p<0.01) and CD86 ^*^(*p*<0.05) after treatment with LPS. This increase was not observed after treatment with LPS in the presence of hBD3 or hBD3 alone, *n*=3 (BMDMs from three separate mice, significance assessed by one-way ANOVA with Tukey post test).

We confirmed that the repressive effects of hBD3 on gene transcription were reflected by inhibitory effects on the resulting gene products. Measurement of IL-6, IL-12p40 and RANTES proteins (the gene products of *Il6*, *Il12a* and *Ccl5*, respectively) after 18 h treatment with KLA or LPS without or with hBD3 reflected the gene expression results. These protein measurements were carried out in both BMDMs (induced from BM by CSF-1) and conventional myeloid dendritic cells (cDC) (induced from BM by GM-CSF) to examine potential differences in hBD3 effect between cells with different backgrounds of cytokine/chemokine environment. Exposure of BMDM to 50 ng/mL of the TLR4 ligands, resulted in a significant increase in IL-6, IL-12p40 and RANTES (Fig. 3A). The presence of 5 μg/mL of hBD3 significantly reduced the induction of these cytokines (Fig. 3A) and IFN-β (Supporting Information Fig. 3). The same inhibitory effects were observed in cDCs (Fig. 3B), despite different basal levels of cytokine (particularly IL-12p40) in the untreated cells.

In addition, the suppression of co-stimulatory molecule gene expression by hBD3 was confirmed using flow cytometric analysis. Co-stimulatory molecules, CD40 and CD86, are often used as a measure of macrophage activation. In agreement with the gene expression data, LPS treatment resulted in the expression of CD40 and CD86 proteins on the cell surface. This co-stimulatory molecule expression was significantly suppressed in the presence of hBD3 (Fig. 3C and D and Supporting Information Fig. 4). These data demonstrate that the repression of *Il6*, *Il12a*, *Ccl5*, *CD40* and *CD86* transcripts by hBD3 is consistent with a reduction in the protein levels as measured by ELISA or flow cytometry.

### hBD3 is associated with a global attenuation of early LPS effects

TLR4 stimulation of BM-derived immune cells results in significant transcriptional changes within 1 h 24, 25. As we have shown that hBD3 is in the cell cytoplasm at this time we sought to determine whether these early transcriptional events are suppressed by hBD3. We have shown that hBD3 inhibition of LPS-induced TNF-α is more complete than hBD3 inhibition of KLA-induced TNF-α (see Fig. 2A), we therefore treated macrophages for 1 h with LPS in an effort to optimize the experiment at this early time point. This also replicates the TLR4 stimulation demonstrated in previous studies 21, 24 which used LPS from *Escherichia coli* serotype 055:B5. The list of genes increased by LPS at 1 h (Supporting Information Fig. 2, list 4) demonstrated a significant positive correlation (Pearson's *r*=0.579, *p*<0.01) with a comparable data set: the 1 h LPS gene expression profile presented by Amit et al. 21. Treatment with LPS in the presence of hBD3 at 1 h again displayed patterns consistent with hBD3 globally attenuating the response to LPS (Supporting Information Fig. 5).

At the 1 h time point only 21 genes were found to be significantly differentially expressed, which is insufficient to generate significant enrichment results using traditional, hard threshold-based methods such as DAVID. However, we were able to seek broader trends in these data using GOrilla 26, a more sensitive approach to GO enrichment analysis that is not dependent on arbitrary thresholds of significance. Using this approach it was found that the enrichment of GO terms in the most altered genes from the 1 h LPS data set reflected the known effects of LPS on macrophage functions. Annotation terms related to the immune response, inflammation and apoptosis were strongly and significantly enriched within the LPS-induced up-regulated genes. Comparison with the data set generated by LPS treatment in the presence of hBD3 (Supporting Information Fig. 2, list 5) revealed that many of the same terms were significantly enriched but fewer genes were involved (Supporting Information Table 2) with some of these being actively repressed. This actively repressed group was evident as 8401 genes, all of which were up-regulated by LPS (positive log FC) but down-regulated by LPS in the presence of hBD3 (negative log FC). We sought to identify significant enrichment of particular pathways among the genes showing the strongest levels of repression using GOrilla. Analysis of GO term enrichment within this sorted list revealed significant terms relating to apoptosis (GO:0006915), immune system process (GO:0002376) and positive regulation of NF-κB transcription factor activity (GO:0051092) (Supporting Information Table 3). The latter group included genes from TLR signaling pathways, such as *Tlr2*, *MyD88* and *IRAK2*, suggesting that hBD3 suppresses TLR signaling components as early as 1 h post-treatment. Findings relating to apoptosis are potentially of interest as hBD3 has been shown to inhibit apoptosis of neutrophils 27.

### hBD3 has no major effects in the absence of LPS

The overall distributions of log FC (Fig. 2B and Supporting Information Fig. 3) for hBD3, LPS and combined hBD3 and LPS treatments show that hBD3 had minimal effects on expression, as hBD3 log FC values more closely approximate zero than the other treatment data sets. No genes were found to be significantly differentially expressed after treatment of BMDM with hBD3 and analysis using the GOrilla method failed to identify any broader enrichment trends among the most altered genes, demonstrating that hBD3 has little measurable effect on gene expression in the absence of LPS. This finding was reflected in the lack of cytokine response in macrophages exposed to hBD3 (Fig. 3A).

### hBD3 inhibits both MyD88 and TRIF signaling

**Figure 4:**
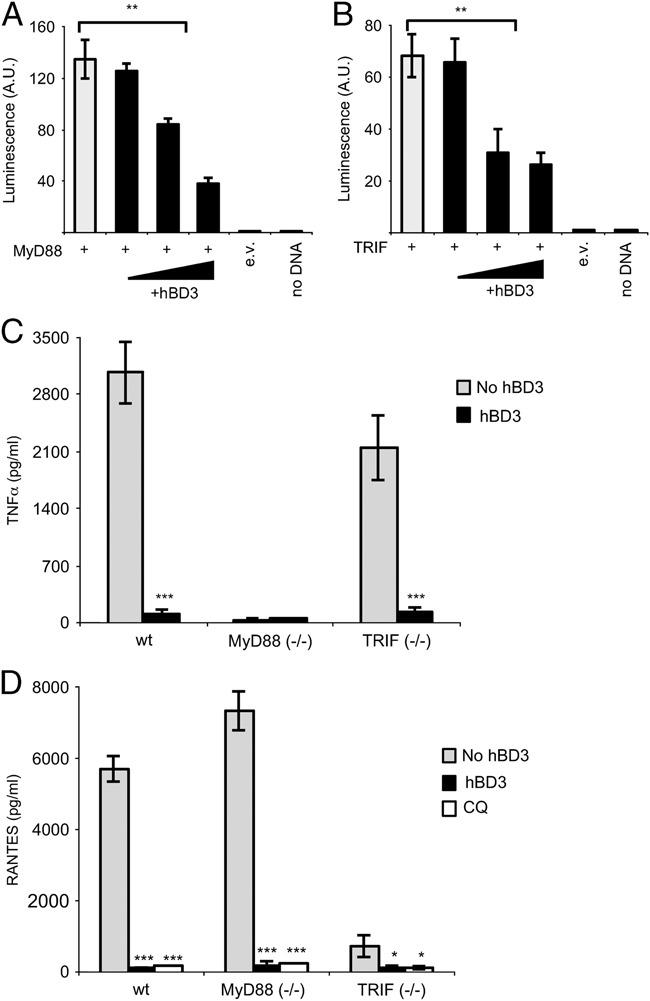
hBD3 inhibits intracellular signaling via both the MyD88 and TRIF pathways. (A, B) HEK 293 cells were transfected with an *NF*-κ*B-luciferase* reporter construct in combination with expression for either, (A) *MyD88* or (B) *TRIF* and 50–200 ng of *hBD3 cDNA* expression construct. *NF*-κ*B* luciferase activity was measured after 18 h. Data were normalized against *tk-*renilla luciferase and expressed as arbitrary units. Data are presented as mean±SEM, significance assessed by unpaired *t*-test, ^**^p<0.01 was calculated by comparing *MyD88* (or *TRIF*) co-transfected with 200 ng empty vector (gray bar) to *MyD88* (or *TRIF*) co-transfected with increasing amounts of *hBD3* plasmid (solid bars). *n*=3 (e.v.=empty vector alone). (C, D) BMDMs from w/t C57BL/6, MyD88^−/−^ and TRIF^−/−^ mice were stimulated with 50 ng/mL LPS in the presence and absence of 5 μg/mL hBD3 or CQ. Levels of (C) TNF-α and (D) RANTES in tissue culture supernatants were measured by ELISA. Data are presented as means±SEM, significance assessed by unpaired *t*-test, ^***^p<0.001, ^*^p<0.05 was calculated by comparing LPS plus hBD3 with LPS alone. *n*=4 (BMDM from four separate mice).

The gene expression profiles of TLR4-activated macrophages in the presence and absence of hBD3, indicated that hBD3 resulted in down-regulation of genes involved in TLR signaling pathways. This suppression is likely to be the cause of the observed down-regulation of NF-κB and inflammatory gene transcription. Downstream of TLR4, LPS activates both MyD88-dependent and independent (TRIF dependent) pathways 28. To further investigate the effects of hBD3 on these pathways, we transiently expressed MyD88 and TRIF in HEK 293 cells with an *NF*-κ*B*-luciferase reporter plasmid (*NF*-κ*B*-*luc*). Transfecting with either *MyD88* or *TRIF* plasmid resulted in expression of MyD88 or TRIF protein and subsequent induction of *Nf*-κ*B-luc* activity (Fig. 4A and B). Both MyD88- and TRIF-induced *NF*-κ*B-luc* activity was inhibited in a dose-dependent manner when *hBD3* was co-transfected at increasing concentrations (50, 100 and 200 ng) (Fig. 4A and B). The concentration of total transfected plasmid was kept constant by using increasing amounts of empty vector as the amount of *hBD3* plasmid decreased. Empty vector had no effect on *NF*-κ*B-luc* signal (Fig. 4A). This demonstrates that hBD3 targets the Myd88 and TRIF pathways downstream of both the ligand (LPS) and the receptor (TLR4).

We further validated this result by examining the ability of hBD3 to suppress an inflammatory response in the absence of MyD88 or TRIF using MyD88^−/−^ and TRIF^−/−^ mutant mice. BMDM from wildtype (w/t) C57BL/6 mice were stimulated with 50 ng/mL LPS to induce TNF-α and RANTES. This induction was significantly suppressed in the presence of hBD3 (Fig. 4C and D). BMDM from MyD88^−/−^ mice did not induce TNF-α in response to LPS, indicating that TNF-α production by LPS is MyD88 dependent, whereas RANTES production is not reduced in the absence of MyD88. In the presence of hBD3, RANTES was inhibited to a similar extent to the inhibition afforded by the endosomal inhibitor chloroquine (CQ) (Fig. 4D). Treatment of TRIF^−/−^ BMDM with LPS resulted in an increase in TNF-α (through the MyD88 pathway) which was inhibited in the presence of hBD3. The response to pI:C in these TRIF null macrophages was abrogated, verifying the inability of these cells to signal through the TRIF pathway. RANTES was only minimally increased by LPS in TRIF^−/−^ BMDM, however this increase was also suppressed in the presence of hBD3 (Fig. 4D). These data show that hBD3 prevents both MyD88 and TRIF signaling.

## Discussion

The current literature on the immunomodulatory activity of defensins and indeed other antimicrobial, cationic amphipathic peptides, such as LL-37, indicates that the function of antimicrobial peptides in immunity is complex and unresolved 9, 14, 15, 29, 30. There is incomplete evidence to suggest either a pro-inflammatory or anti-inflammatory role, and it may be that like LL-37, β-defensins are multifunctional modulators of the immune response, demonstrating pro-inflammatory effects at high concentrations but anti-inflammatory effects at lower concentrations 31. It is possible that hBD3 is induced at high levels at the site of pathogen entry, causing a pro-inflammatory response involving chemoattraction of macrophages and other immune cells. As the danger is neutralized and levels of hBD3 and other pro-inflammatory molecules decrease, hBD3 may then have a role in resolving inflammation.

In previous work, we focused on the effects of hBD3 on protein levels of TNF-α and IL-6 and showed no measurable increase in these cytokines in hBD3-treated macrophages 15. The gene expression analyses carried out in the present study allowed a more comprehensive investigation of the effects of hBD3 at the transcription level. Enrichment analysis of all differentially expressed genes, failed to identify any pro-inflammatory markers after hBD3 treatment. In contrast, hBD3 has been shown to induce cell surface co-stimulatory molecule expression in human monocytes and myeloid DCs 9. We observed no increase in co-stimulatory molecules after hBD3 treatment and in fact observed a reduction in LPS-induced CD40 and CD86 at both the gene transcript and protein level (Figs. 2C, 3C and D). We wondered whether the contrasting results may be explained by structural integrity of the hBD3 molecule. We show that in the absence of LPS neither canonically folded hBD3 nor hBD3 without disulfide bonds induce a pro-inflammatory cytokine response. However, when testing the ability of hBD3cys-ser to inhibit the effects of LPS we discovered that hBD3cys-ser in the presence of LPS induced a pro-inflammatory response significantly higher than that of LPS alone.

It is possible that the work in vitro by others reflects the use of an LPS-contaminated or non-canonically folded form of the peptide. In vivo defensin molecules may be destabilized by reduction in the disulfide bonds and recently it was shown that reduced hBD1 demonstrated enhanced antimicrobial activity 32. We have previously demonstrated the ability of hBD3 to limit the pro-inflammatory effect of LPS in mice, indicating that disulfide bond reduction is unlikely to occur in vivo 15.

Gene expression analysis indicated that inhibition by hBD3 affected gene transcription, therefore hBD3 does not act on the available TNF-α/IL-6 reservoir of protein already present in the cell. This provides evidence that hBD3 interferes with LPS signaling at some point between LPS-TLR4 receptor binding and transcription factor activation. GO term enrichment analysis of transcripts at 1 h shows that hBD3 down-regulates components of TLR signaling pathways. This in turn leads to the observed suppression of chemokine and cytokine transcripts at 6 h, and also at this time many components of TLR signaling remain down-regulated (Fig. 2C).

Downstream of TLR4, LPS utilizes both MyD88-dependent and TRIF-dependent signaling pathways. In macrophages, MyD88 is recruited to activate TLR4 by MAL and results in early NF-κB and AP-1-mediated induction of pro-inflammatory cytokines 33. Activation of TLR4 also promotes receptor endocytosis with Rab11a, which causes a release of TLR4 for subsequent interaction with TRAM and TRIF 34. There is then a later wave of NF-κB, AP-1 and IRF3 activation and subsequent induction of IFN-regulated genes 35. At 1 h after LPS treatment, hBD3 is very effective at inhibiting the effect of LPS where activation is assumed to be via the early MyD88-dependent pathway, implying that hBD3 targets MyD88 signaling. This result is supported by the fact that the hBD3 inhibitory effect is evident in cells transfected with MyD88-expression construct and also in LPS-stimulated macrophages lacking TRIF (therefore only signaling via MyD88). However, hBD3 also suppresses TRIF signaling in both HEK 293 cells expressing TRIF and in LPS-stimulated MyD88-deficient macrophages. hBD3 must therefore target a signaling component shared by both the MyD88 and TRIF pathways or target components in both pathways. The downstream components of both the MyD88 and TRIF pathways are currently being investigated as potential targets of hBD3 action.

We propose that hBD3 enters immune cells and prevents activation of a pro-inflammatory response by specifically binding to component(s) of TLR signaling pathways. A similar mechanism has recently been described as an immune evading mechanism for *Vaccinia virus* which encodes a protein which prevents TLR signaling by binding the multiple cytoplasmic Toll/IL-1R (TIR) domain-containing adaptor proteins, MAL and TRAM 36. These researchers found that the inhibitory effects were specific to TLR4. A comparison of hBD3 and the active component of the *Vaccinia virus* protein (VIPER) revealed no sequence similarities between the two peptides. We are currently continuing to investigate the protein targets of hBD3 to determine the point at which hBD3 prevents TLR signaling.

Components of TLR pathways are potential therapeutic targets in inflammatory and autoimmune disease 37. Over activation of TLR4 can give rise to endotoxic shock often resulting in sepsis and death. In addition, TLR4 can respond to endogenous damage-associated signals and give rise to sterile inflammation, a mechanism recently suggested for atherosclerosis and Alzheimer's disease 38. Among the population the variability in β-defensins copy number, specifically hBD3, may result in differing susceptibility of some individuals to disease-induced inflammation 39, 40. Clarification of the molecular basis of the interaction of hBD3 with TLR inflammatory pathway may be of direct clinical relevance, and in addition shed light on the role of hBD3 in inflammatory disease.

## Materials and methods

### Reagents

Ultra-pure lipopolysaccharide (LPS) from *E. coli* 0111:B4, lipoteichoic acid (LTA), Pam3CSK4, polyI:C, R848 and IFN-γ were purchased from InvivoGen (San Diego, USA), di[3-deoxy-d-manno-octulosonyl]-lipid A (KDO_2_-Lipid A) from Avanti Polar Lipids (Netherlands). CQ was purchased from Autogen Bioclear UK (Wiltshire, UK). M-CSF and GM-CSF and ELISA DuoSets were obtained from R&D Systems (Abington, UK). hBD3 (GIINTLQKYYCRVRGGRCAVLSCLPKEEQIGKCSTRGRKCCRRKK) was purchased from Peptides International or synthesised by solid-phase peptide synthesis as described.

### Solid-phase peptide synthesis and oxidative folding

All peptides were produced on an Applied Biosystems 433A peptide synthesizer by standard Fmoc (fluorenylmethyloxycarbonyl chloride) chemistry (full details are in Supporting Information).

### Cell-lines and primary cell preparation

RAW264.7 cells were maintained in DMEM (GIBCO) containing 10% fetal bovine serum (FBS), essential amino acids and antibiotics. Balb/c and C57 Black/6 mice were obtained from Charles River (UK) and MyD88^−/−^ and TRIF^−/−^ were a kind gift from David Gray (University of Edinburgh). Primary cells were generated from femur BM and grown for 7–10 in DMEM containing 10% fetal bovine serum and either 20 ng/mL M-CSF to generate macrophages or 20 ng/mL GM-CSF to generate cDCs. Cells were seeded at 2×10^5^ into 48-well plates and grown without growth factor for 24 h prior to treatment. Replicate experiments were performed with separate primary cell preparations from at least three mice for each experiment.

### Cell culture for live cell image analysis

RAW264.7 cells were cultured overnight in 24-well glass-bottomed plates coated with poly-ethylene imine (Sterilin, London, UK) in DMEM (supplemented as above). hBD3 labeled with the TAMRA fluorochrome (HBD3^TAMRA^) was added to the media at 5 μg/mL just prior to image analysis.

### Image analysis

Live cell image analysis was carried out using a Zeiss Axiovert 200 fluorescence microscope (full details are in Supporting Information). Z-stacks were captured over a distance of 30 μM at 0.2 μM per slice. An image was selected from the center of the stack to show the distribution of HBD3^TAMRA^ within the cell. Images have been processed with a γ adjustment of 0.4 to enhance the midrange values.

### Microarray protocol and analysis

RNA was extracted, with RNA bee (AMS Biotechnology, Abingdon, UK) using standard methods, from BMDM (three biological replicates per treatment) treated with hBD3 (5 μg/mL), LPS or KLA (50 ng/mL), a combination of hBD3 and LPS or KLA or untreated. Amplified, biotinylated cRNA was synthesized using Illumina® TotalPrep Amplification Kit (Ambion, Austin, Texas) and microarray experiments were performed using 2 Illumina MouseWG-6 v2.0 BeadChips for 6 h treatments, and Illumina MouseRef-8 v2.0, for 1 h treatments. The data sets are available from Gene Expression Omnibus (GEO) accession numbers GSE25870 and GSE25871. Briefly, the analysis consisted of standard normalization procedures, followed by a widely used linear model approach to the detection of differential expression, coupled with a multiple testing correction. Full details of the microarray analysis are in Supporting Information.

### Cell treatment and ELISA

Cells were treated with LPS (50 ng/mL) or KLA (50 ng/mL), in the presence or absence of hBD3 (5 μg/mL) in serum-free media then incubated at 37°C, 5% CO_2_ for 18 h. Cell viability was measured using TACS_TM_ MTT assay (R&D Systems). Where appropriate, cells were pre-incubated with hBD3 for the required time, followed by two washes with PBS before treating with LPS in conditioned media. Supernatants were collected and centrifuged then levels of TNF-α, IL-6, IL-12p40 and RANTES were measured using DuoSet ELISA (R&D Systems) according to manufacturer's instructions. Where appropriate, statistical significance was determined by an unpaired *t*-test using the GraphPad software. For all statistical analyses, *p*<0.05 was considered significant. Values are expressed as mean±SEM.

### Cell transfection and luciferase detection

Full-length *hBD3* cDNA was amplified with restriction fragment linkers (NotI and XhoI) and cloned into expression vector *pcDNA3.1* (Invitrogen, UK). HEK293 cells were co-transfected with NF-κB luciferase plasmid and MyD88 (EBG6P-MyD88; a kind gift from Prof. Cohen) or TRIF (pflag-CMV2 TRIF; a kind gift from Prof. Bowie) expression plasmids in combination with increasing amounts of hBD3 expression plasmid. Total transfected DNA was kept constant with pcDNA 3.1 empty vector. Dual-luciferase kits (Promega) were used for subsequent analysis. NF-κB Firefly luciferase activity was normalized for cell transfection with *tk*-renilla luciferase co-transfection.

### Co-stimulatory molecule detection

Expression of CD40 and CD86 on the surface of BMDM following treatment with LPS and/or hBD3 was assessed by flow cytometry. Cells were incubated for 20 mins at room temperature with Mouse BD Fc Block™ (BD Biosciences, Oxford, UK) then for 40 mins at 4°C in the dark, with APC-conjugated rat anti-mouse antibodies against CD40 or CD86 (eBioscience, San Diego, CA). Appropriate isotype controls were used. Flow cytometric data collection was carried out with a BD FACSARIAII SORP (BD Biosciences, Oxford, UK), using a 640 nm laser (670/14 nm bandpass filter). Data were analyzed using the FlowJo Version 7.5.5 (Treestar, Olten, Switzerland) and statistical analysis carried out using one-way ANOVA with Tukey post test.

## Abbreviations

cDC: conventional myeloid DC
CQ: chloroquine
hBD3cys-ser: hBD3 where the six cysteine residues are replaced by serines
KLA: KDO_2_-Lipid A
logFC: log fold change

## Notes

Detailed facts of importance to specialist readers are published as ”Supporting Information”. Such documents are peer-reviewed, but not copy-edited or typeset. They are made available as submitted by the authors.
