# Supplementary material for: Human β-defensin 3 affects the activity of pro-inflammatory pathways associated with MyD88 and TRIF
Source: Eur J Immunol. 2011 Aug 2;41(11):3291–300. doi: 10.1002/eji.201141648 (PMC3494976; doi:10.1002/eji.201141648)
Supplement: Supplementary file 1 [file eji0041-3291-SD1.zip › Supporting_information.pdf]

European Journal of Immunology

Supporting Information

for

DOI 10.1002/eji.201141648

Human β-defensin 3 affects the activity of pro-inflammatory pathways associated with MyD88 and TRIF

Fiona Semple, Heather MacPherson, Sheila Webb, Sarah L. Cox, Lucy J. Mallin, Christine Tyrrell, Graeme R. Grimes, Colin A. Semple, Matthew A. Nix, Glenn L. Millhauser and Julia R. Dorin

Supplementary Figure 1

Fluorescence observed in the RAW264.7 cells is due to the uptake of HBD3^{TAMRA} and not free fluorochrome.

In order to assess whether the fluorescence observed in the cells was due to the uptake of HBD3^{TAMRA} or TAMRA alone RAW 264.7 cells were exposed for 10 minutes to equimolar amounts of either HBD3^{TAMRA} (Panel on left) or TAMRA alone (Panel on right) for 10 minutes after which time the cells were fixed and imaged as outlined in the materials and methods. The images captured for the two different treatments (HBD3^{TAMRA} or TAMRA alone) were done during the one imaging session using the same exposure times. The raw data was then displayed for both images in the same window. The background was then removed from the final montage using both brightness and contrast controls with no gamma adjustment ensuring that both images were treated the same. The panel on the left shows that the HBD3^{TAMRA} enters the cells within 10 minutes of exposure whereas the panel on the right shows that the TAMRA alone does not enter the cell indicating that the uptake is specific to the HBD3^{TAMRA}. TAMRA shown in Red. DAPI shown in blue. Scale bar = 10μm.

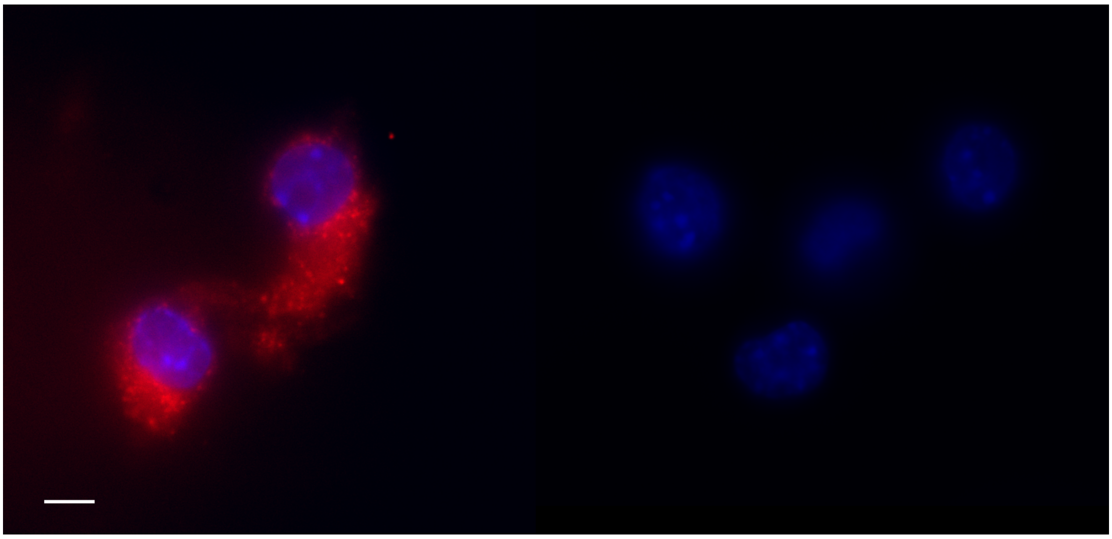

Supplementary Figure 2. Microarray data lists showing fold change values for all genes in the Illumina array (see separate file)**.**

Supplementary Figure 3. BMDM were treated with 50ng/ml LPS in the presence or absence of 5μg/ml hBD3 for 18hr. IFNβ levels in the cell culture supernatants were measured by ELISA, n=3 (BMDM from 3 separate mice). Figure shows means +/- SEM, significance assessed by unpaired t-test, *p<0.05 was calculated by comparing LPS plus hBD3 to LPS alone.

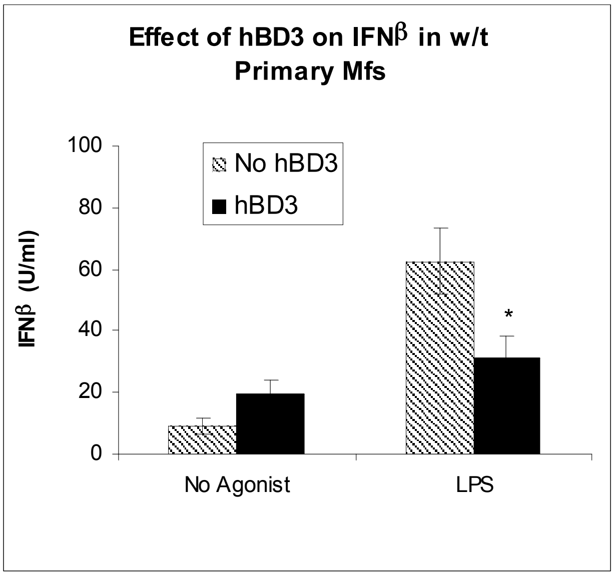

Supplementary Figure 4. BMDM were treated with 50ng/ml LPS in the presence and absence of 5μg/ml hBD3. Cell surface expression of CD40 and CD86 was assessed by flow cytometry. Raw data show fluorescence shift after LPS stimulation, which is not apparent after treatment with combined LPS and hBD3 (representative sample of n=3).

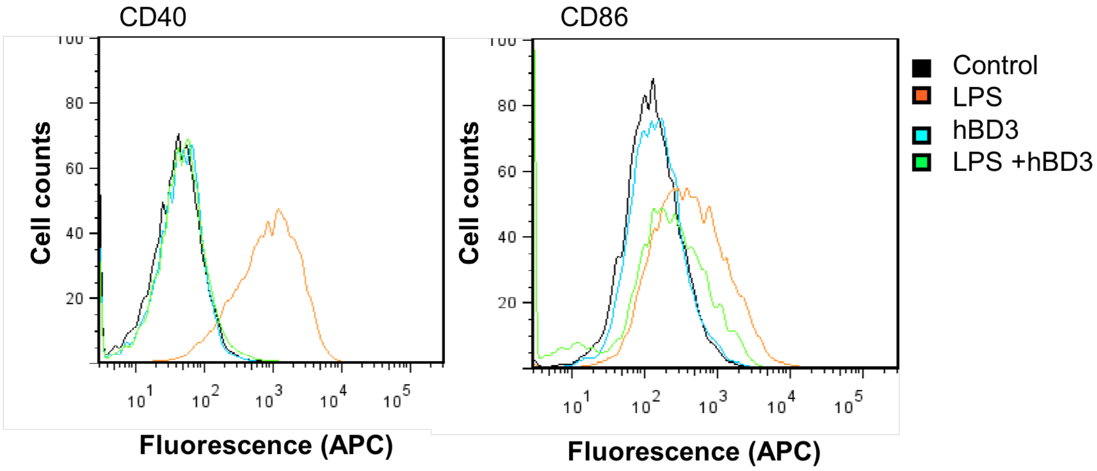

Supplementary Figure 5. Global gene expression changes in BMDM treated for 1 hour with 50ng/ml LPS in the presence and absence of 5μg/ml hBD3. Each box represents the interquartile range centred upon the median (and containing 50% of the values), the whiskers denote 1.5 times the interquartile range, and the circles represent outlier values beyond the whiskers. In each case the distribution of logFC values (y-axis) for all genes on the array are shown relative to untreated cells. Each circle on the graph represents a gene on the array.

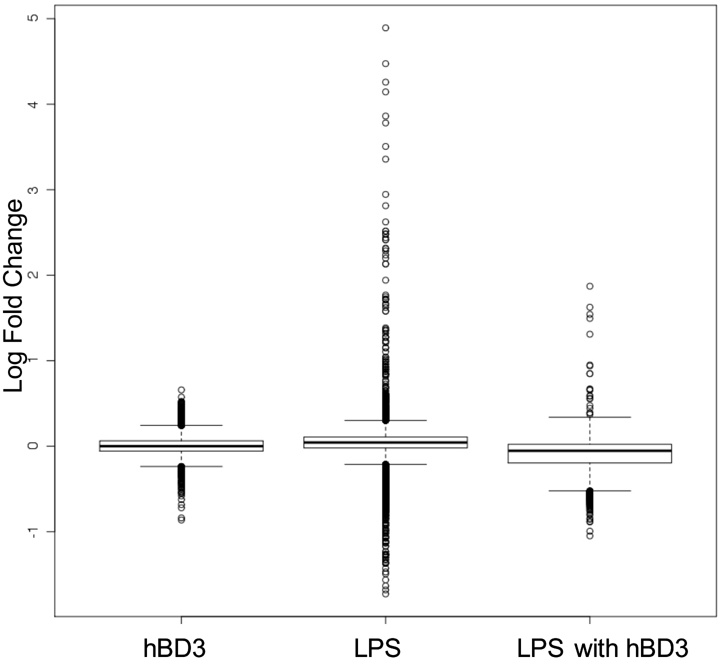

Table S1: KEGG enrichment among genes suppressed by hBD3

Term	Number of genes in pathway	Adjusted p value (Benjamini & Hochberg method)
Toll-like receptor pathway	23	p<0.001
NOD-like receptor signalling pathway	17	p<0.001
Jak-STAT signalling pathway	23	p<0.05
Adipocytokine signalling pathway	14	p<0.05
MAPK signalling pathway	32	p<0.05
Cytokine-cytokine receptor interaction	30	p<0.05
ErbB signalling pathway	15	p<0.05
Cytosolic DNA sensing pathway	11	p<0.05

Table S2: GO terms enriched in genes upregulated in response to LPS shown to be suppressed with the addition of hBD3. All GO terms found to be significantly enriched in upregulated genes with both LPS treatment and combined LPS and hBD3 treatment are listed with the corrected enrichment p-values and the numbers of genes (n) involved in each dataset.

		**LPS**		**LPS with hBD3**	
**GO term**	**Description**	**p**	**n**	**p**	**n**
GO:0006954	inflammatory response	1.54e-09	16	1.39e-05	8
GO:0050900	leukocyte migration	5.53e-05	4	1.88e-05	4
GO:0009611	response to wounding	7.07e-09	17	1.16e-04	8
GO:0006952	defense response	1.02e-09	24	0.000273	13
GO:0006955	immune response	8.61e-11	20	0.000756	9
GO:0030593	neutrophil chemotaxis	0.00115	4	0.000826	3
GO:0051239	regulation of multicellular organismal process	1.05e-07	25	0.0011	12
GO:0070887	cellular response to chemical stimulus	2.84e-05	12	0.00145	4
GO:0002376	immune system process	8.61e-12	28	0.00159	5
GO:0051716	cellular response to stimulus	4.07e-06	21	0.00213	5
GO:0030595	leukocyte chemotaxis	0.0028	6	0.00342	3
GO:0045941	positive regulation of transcription	4.18e-06	18	0.00415	8
GO:0060326	cell chemotaxis	0.00438	6	0.00421	3
GO:0050793	regulation of developmental process	5.02e-08	27	0.00569	10
GO:0010628	positive regulation of gene expression	1.87e-06	19	0.00569	8
GO:0016477	cell migration	0.0184	4	0.0066	4
GO:0045935	positive regulation of nucleobase, nucleoside, nucleotide and nucleic acid metabolic process	1.68e-07	16	0.00938	8
GO:0048870	cell motility	0.0328	4	0.0105	4
GO:0048522	positive regulation of cellular process	1.57e-12	44	0.0108	13
GO:0050794	regulation of cellular process	1.89e-06	61	0.011	490
GO:0010557	positive regulation of macromolecule biosynthetic process	5.59e-07	20	0.0129	8
GO:0051173	positive regulation of nitrogen compound metabolic process	5.86e-10	23	0.0129	8
GO:0031328	positive regulation of cellular biosynthetic process	2.19e-08	22	0.0206	8
GO:0009891	positive regulation of biosynthetic process	3.52e-10	23	0.0228	8
GO:0065007	biological regulation	1.78e-05	69	0.0352	53
GO:0043065	positive regulation of apoptosis	4.08e-06	21	0.0404	7
GO:0043068	positive regulation of programmed cell death	6.48e-07	22	0.0420	7
GO:0048518	positive regulation of biological process	4.20e-12	46	0.0464	13
GO:0010942	positive regulation of cell death	1.02e-06	22	0.0477	7
GO:0009605	response to external stimulus	0.039	8	0.0487	5
GO:0042379	chemokine receptor binding	3.16e-05	8	0.00427	5
GO:0005125	cytokine activity	3.28e-10	17	2.90e-07	9
GO:0005126	cytokine receptor binding	3.61e-09	14	7.08e-05	7
GO:0005102	receptor binding	2.10e-06	25	0.0035581	14
GO:0008009	chemokine activity	2.46e-05	8	0.0037	5
GO:0005615	extracellular space	1.01e-06	20	3.21e-05	14
GO:0044421	extracellular region part	0.000272	21	8.52e-05	80

Table S3: LPS response pathways attenuated in the presence of hBD3 after 1hr. All GO terms found to be significantly enriched in the subset of genes up-regulated with LPS treatment but down-regulated with LPS treatment in the presence of hBD3 are listed with the corrected enrichment p-values and the numbers of genes (n) involved.

GO Term	Description	P	N
GO:0006915	apoptosis	2.864994e-05	25
GO:0012501	programmed cell death	3.946558e-05	25
GO:0002376	immune system process	0.03348246	10
GO:0051092	positive regulation of NF-kappaB transcription factor activity	0.03359752	7
GO:0044260	cellular macromolecule metabolic process	0.03423035	286
GO:0045944	positive regulation of transcription from RNA polymerase II promoter	0.04038606	7
GO:0045893	positive regulation of transcription, DNA-dependent	0.04337762	8
GO:0051254	positive regulation of RNA metabolic process	0.04493093	8
GO:0008219	cell death	0.0003722191	25
GO:0016265	death	0.000419969	25
GO:0051173	positive regulation of nitrogen compound metabolic process	0.001996291	8
GO:0009893	positive regulation of metabolic process	0.003762462	11
GO:0009891	positive regulation of biosynthetic process	0.00684607	10
GO:0031325	positive regulation of cellular metabolic process	0.01058552	8
GO:0048522	positive regulation of cellular process	0.01116082	14
GO:0045935	positive regulation of nucleobase, nucleoside, nucleotide and nucleic acid metabolic process	0.02375989	9
GO:0044424	intracellular part	4.9766e-11	672
GO:0043231	intracellular membrane-bounded organelle	3.6058e-08	514
GO:0043227	membrane-bounded organelle	3.83675e-08	514
GO:0043226	organelle	7.5245e-07	545
GO:0043229	intracellular organelle	1.2516e-06	543
GO:0005634	nucleus	0.00034568	306
GO:0005737	cytoplasm	0.00163155	239

Supplementary Methods

Solid Phase Peptide Synthesis and Oxidative Folding

All peptides were produced on an Applied Biosystems 433A peptide synthesizer by standard Fmoc (fluorenylmethyloxcarbonyl chloride) chemistry. Amino acids were purchased from NovaBiochem and were assembled on Rink-amide-MBHA resin. Pre-activated Fmoc-Cys(Trt)-OPfp was used to avoid enantiomerization. Fmoc protecting groups were removed using a 1% 1,8-diazabicyclo[5.4.0]undec-7-ene (DBU) / hexamethyleneimine (HMI) mixture in N, N,-Dimethyl Formamide (DMF) and 4 equivalents amino acid/ O-Benzotriazole-N,N,N',N'-tetramethyl-uronium-hexafluoro-phosphate (HBTU)/ N, N-Diisopropylethylamine (DIAE) mixture was used for coupling (except Pre-activated Fmoc-Cys(Trt)-Opfp, which was only coupled in DMF solution). The N-terminal labeling of peptides with fluorescent dye was performed on resin-bound peptide using 4 equivalents of 5,6 carboxytetramethylrhodamine succinimidyl ester (5(6)-TAMRA SE) and 6 equivalents of DIAE in DMF, incubating for 2 hours. Cleavage was performed in a Trifluoroacetic acid (TFA)/ Triisopropylsilane (TIS) /1, 2-Ethanedithiol (EDT)/ Phenol (90:4:4:2) mixture for 90min. Oxidative folding was achieved by dissolving peptides to a concentration of 0.1mg/mL in folding buffer (0.5-1.0M Guanidine hydrochloride (GuHCl), 0.1M Tris, 1mM glutathione (GSH), 0.1mM oxidized glutathione (GSSG), pH 8.5) and stirring for 48 hours. Folding was monitored by HPLC, which, in each case, revealed one major species that was used in subsequent experiments. Folding procedures were developed to give the correct HBD3 structure, as verified previously by nuclear magnetic resonance structure determination The folded products were purified by C18 column HPLC and identified as fully oxidized peptides by mass spectrometry.. Quantitative concentrations were determined with amino acid analysis at the molecular structure facility at UC Davis.

Image Analysis

Live cell image analysis was carried out using a Zeiss Axiovert 200 fluorescence microscope equipped with 100x/1.4 plan apochromat objective (Carl Zeiss, Welwyn, UK), Lambda LS 300W Xenon source with liquid light guide, excitation and emission filterwheels (Sutter Instrument, Novato, CA) with #86000 Sedat quad set (Chroma Technology Corp., Rockingham, VT), ASI PZ2000 3-axis XYZ stage with integrated

piezo Z-drive (Applied Scientific Instrumentation, Eugene, OR), and Photometrics Coolsnap HQ2 CCD camera (Roper Scientific, Tucson, AZ). The system is equipped with a Solent Scientific incubation chamber with CO^2 enrichment (Solent Scientific, Segensworth, UK). Z-stacks were captured over a distance of 30μM at 0.2μM per slice. An image was selected from the centre of the stack to show the distribution of $HBD3^{TAMRA}$ within the cell.

Microarray analysis

Raw data from Beadstudio was imported into R using the bioconductor package Beadarray (Dunning, 2007) and Quantile normalization was applied to summarized probe expression level to correct for systematic differences in expression level across/between chips. Normalized expression data were analyzed with the Limma bioconductor package (Smyth, 2005) using a two-stage linear statistical model to find differentially expressed genes between treatment groups. P-values were adjusted for multiple testing using the Benjamini and Hochberg method. Probes with an adjusted p value of < 0.05 were selected as differentially expressed.

GO enrichment of differentially expressed genes was carried out using the online Functional Annotation Tool DAVID (Database for Annotation, Visualisation and Integrated Discovery) and p-values were adjusted for multiple testing using the Benjamini and Hochberg method. Additional, complementary analysis was carried out using GOrilla to discover further enriched GO terms with a more flexible thresholding approach. GOrilla finds the point in the list at which the most significant enrichment is obtained for a given GO term, and then calculates the significance of this enrichment relative to the remainder of the ranked gene list (Eden, 2009). All datasets were processed using GOrilla (for the three GO hierarchies: biological process, molecular function, cellular component) in each case examining the results from ranked lists of genes sorted in an ascending (highest logFC at top) and also a descending (lowest logFC at top) order. This identifies GO terms enriched in the genes most positively and also negatively differentially expressed genes. Enrichment p-values were Bonferroni corrected and reported if < 0.05.

Supplementary Movie Legend

RAW264.7 cells were exposed to 5μg/ml hBD3^{TAMRA} just prior to live cell image analysis using a Zeiss Axiovert 200 fluorescence microscope equipped as outlined in supplementary materials and methods. Z-stacks were captured over a distance of 30μM at 0.2μM per slice every 10 minutes over a 2 hour period. The optimal z-plane from each stack at each time point was selected and sequentially displayed to show the accumulation of hBD3^{TAMRA} within the cell over the 2 hour period.

References

Smyth, G. K. Limma: linear models for microarray data. In: **Gentleman, V., Carey, S., Dudoit, R., Irizarry and W. Huber, W.** (Eds) Bioinformatics and Computational Biology Solutions using R and Bioconductor. R., Springer, New York, 2005.

Dunning, M.J., Smith, M.L., Ritchie, M.E., Tavaré, S. Beadarray: R classes and methods for Illumina bead-based data. *Bioinformatics* 2007. **23**: 2183-2184.

Eden, E., Navon, R., Steinfeld, I., Lipson, D. and Yakhini, Z. GOrilla: a tool for discovery and visualization of enriched GO terms in ranked gene lists. *BMC.Bioinformatics.* 2009. **10**: 48.

Benjamini, Y., and Hochberg, Y. Controlling the false discovery rate: a practical and powerful approach to multiple testing. *J Roy Statist Soc Ser B (Methodological)* 1995. **57**: 289-300.
